# Analysis of transcriptome and metabolome characteristics of blood in yaks at different reproductive stages

**DOI:** 10.3389/fvets.2025.1633877

**Published:** 2025-10-17

**Authors:** Yandong Kang, Jie Pei, Lin Xiong, Xiangying Kong, Fujun Liu, Yuqing Zhou, Shengbin Shang, Yulong Feng, Haiqin Li, Xiaolei Wu, Min Chu, Shoubao Zhao, Xian Guo

**Affiliations:** ^1^Key Laboratory of Yak Breeding of Gansu Province, Lanzhou Institute of Husbandry and Pharmaceutical Sciences, Chinese Academy of Agricultural Sciences, Lanzhou, China; ^2^Key Laboratory of Animal Genetics and Breeding on Tibetan Plateau, Ministry of Agriculture and Rural Affairs, Lanzhou, China; ^3^Haibei Tibetan Autonomous Prefecture Agriculture and Animal Husbandry Comprehensive Service Center, Haibei, China; ^4^Yushu Tibetan Autonomous Prefecture Agriculture and Animal Husbandry Cooperative Economy Service Center, Yushu, China; ^5^Haibei Animal Husbandry Science and Technology Demonstration Park Management Committee, Haibei, China; ^6^Yak Breeding and Promotion Service Centre of Qinghai Province, Xining, China; ^7^Qinghai Vocational and Technical Institute of Animal Husbandry and Vet, Xining, China

**Keywords:** yak, reproduction, transcriptome, metabolome, blood

## Abstract

The reproductive physiology of yaks differs significantly from that of other cattle breeds due to late sexual maturity, low fecundity and short estrus time. How to improve the reproductive efficiency of yaks has become the main research content and goal of yak reproduction technology. In this study, we collected blood samples from adult female yaks (4–8 years old) during different reproductive periods, including the period of anestrus (Y-A), estrus (Y-E) and pregnancy (Y-P), and investigated the changes of RNA expression and steroid hormone levels in yaks during different reproductive periods by using RNA-seq and target metabolomics, and screened for the genes and regulatory pathways. DEGs such as *PDK4*, *ALAS2*, *GLP1R*, *SLC25A39*, *PGAP6*, *FOS*, *CD36*, *MMP9* and *BCL-6* were identified to play key roles in ovarian function, follicular development, hormone homeostasis and energy metabolism. Functional annotation and enrichment analysis indicated that DEGs were involved in ovarian angiogenesis, hormone synthesis and follicular development. In order to reveal the deep interaction between steroid hormone metabolism and gene expression, the weighted gene co-expression network analysis (WGCNA) method was used. It was found that *SLC25A39* may affect glucocorticoid homeostasis and physiological readiness by regulating energy metabolism during anestrus, *MARCHF2* and DHEA may be closely related to reproductive hormone fluctuation and system activation during estrus, glucocorticoid down-regulation in pregnancy and maintenance of hormone homeostasis and regulation of immune tolerance by DHEA. The results of this study provide a theoretical basis for improving the reproductive performance of yaks and further analysing the reproductive characteristics of yaks.

## 1 Introduction

Yak (*Bos grunniens*) is a special breed of cattle mainly distributed in the Qinghai–Tibetan Plateau region, which has received widespread attention for its adaptability to harsh environments, cold and drought tolerance, and high resistance to adversity (1, 2). Yak industry is one of the important animal husbandry industries in the Tibetan Plateau region of China, and in yak industry, breeding production is a key link, which affects the development and economic benefits of the whole industry. Yaks show unique survival adaptability in the extreme high-altitude environment, but their reproductive performance is limited by a series of intrinsic physiological characteristics and external environmental factors (3). Specifically, this is reflected in the long breeding cycle, inconsistent estrous cycle, obscure estrous performance and low conception rate, which poses a serious challenge to the large-scale and intensive development of yak breeding industry.

Blood is a more readily available biological fluid in living organisms and, in contrast to other tissues, blood samples can be sampled repeatedly over time without altering the physiological state of the animal (4). Transcriptomics analysis is a powerful tool for identifying gene expression signatures (5, 6), as a highly accessible source of information, blood is a major conduit for nutrient, cellular and analytical signals. Therefore, the use of blood transcriptomics can be used to detect changes in the physiological state of livestock due to health, reproductive status, diet, nutrition or stress or to develop new biomarkers for infectious diseases (7). Blood metabolomics is a method of assessing the physiological status and reproductive performance of animals by monitoring and analysing metabolites in their blood. It has been shown that the concentration of specific metabolites in the blood correlates with the fertility and length of gestation of the animal. By monitoring metabolites in the blood, it is possible to understand the physiological status, reproductive performance and nutritional requirements of animals during pregnancy (8, 9).

The combination of two technologies, blood transcriptome and blood metabolome, provides more comprehensive information and helps to gain a deeper understanding of the interrelationship between gene expression and metabolite levels in the blood. By analysing the correlation between the two technologies, it is feasible to gain deeper insights into biological processes or disease states, identify potential therapeutic targets or biomarkers, and understand the interaction mechanisms between gene expressions and metabolites, thereby providing more accurate information for disease research and facilitating molecular breeding efforts (10, 11). By comprehensively analysing the transcriptomic and metabolomic data of yak ovarian tissues, the researchers revealed the potential mechanism of N-carbamylglutamate (NCG) on the follicular development process in yaks, which provided valuable insights into the metabolite changes and the regulation of follicular development during the estrous period in yaks (12). In addition, transcriptomic and metabolomic analyses of body fluids from female yaks in estrus and non-estrus conditions revealed the accumulation of metabolites such as amino acids, steroids and organic acids, as well as changes in the expression of key genes, such as miR-129, during estrus in yaks, which provided preliminary insights into the mechanisms of estrus in yaks (11).

In order to study the changes of RNA expression and steroid hormone levels in yak blood during different breeding periods, we screened differentially expressed genes and related regulatory pathways. Whole blood transcriptome sequencing was used to identify differential transcripts in yak blood during estrus and before and after estrus, and target metabolomes were used to identify differential compounds in yak blood during estrus and before and after estrus. The aim of this study is to provide theoretical support for the identification of estrus and early pregnancy diagnosis during yak reproduction through multi-omics analysis, which is of practical significance for improving the calving rate of yaks.

## 2 Materials and methods

### 2.1 Estrus synchronization

Female yaks in good physical condition, with normal reproductive function, free of reproductive tract diseases, and provided with good husbandry management were examined rectally to ensure that there were no female yaks that had been impregnated due to natural mating under grazing conditions, as well as those that had not yet given birth. The simultaneous estrus programme used a combination of progesterone pessary + PGF_2α_ + GnRH, which was firstly buried with a progesterone pessary (Institute of Animal Husbandry Science and Technology, Aba Tibetan and Qiang Autonomous Prefecture, Sichuan Province, China), and the day of pessary release was set as day 0; the pessary was removed on day 7 and an intramuscular injection of sodium cloprostenol (PGF_2α_ 0.4 mg/head) (Ningbo Sansheng Biotechnology Co., Ltd., Zhejiang Province, China) was given; an injection of gona/gonadrin (GnRH 100 μg/head) (Ningbo Second Hormone Factory, Zhejiang Province, China) was given on day 9; and estrus was identified on day 10, together with her timed insemination. The artificial insemination was carried out with reference to the Agricultural Industry Standard of the People’s Republic of China “Technical Procedures for Artificial Insemination of Yaks” (NY/T 3979-2020).

### 2.2 Sample collection

Blood samples were tracked during anestrus (Y-A), estrus (Y-E) and pregnancy (Y-P). Whole blood was collected for transcriptome sequencing using anticoagulated (EDTA-containing) blood collection tubes, with 250 μL whole blood + 750 μL TRIzol LS per tube, and whole blood was collected for metabolome sequencing using anticoagulated (EDTA-containing) blood collection tubes, with 1 mL of blood per tube, and the blood samples were processed and stored in liquid nitrogen immediately after collection. To ensure that the blood samples were collected at the correct stage, the blood of six cows in their second year of calving was selected for sequencing. The samples were collected from the artificially inseminated herd of Yak Breeding and Promotion Service Centre of Qinghai Province (E101°22′, N37°15′), and all the animals were adult yaks aged 4–8 years.

### 2.3 Total RNA extraction and transcriptome sequencing analysis

Total RNA was extracted from blood samples using Trizol LS reagent (Thermo Fisher Scientific) according to the manufacturer’s instructions. Briefly, samples were homogenized, mixed with chloroform, and centrifuged to separate phases. The aqueous RNA-containing layer was collected, and RNA was precipitated with isopropanol, washed with 75% ethanol, and finally dissolved in nuclease-free water. RNA integrity and concentration were assessed, and qualified samples were stored at −80 °C for subsequent analysis.

RNA sequencing libraries were prepared from 5 μg of total RNA using the NEBNext Ultra II RNA Library Prep Kit (New England Biolabs, United States), in accordance with the manufacturer’s instructions. Final library quality was assessed using an Agilent Bioanalyzer, revealing an average insert size of 300 ± 50 bp. Paired-end sequencing (2 × 150 bp) was conducted on an Illumina Novaseq 6000 platform. The downstream raw data format was Fastq, and the Cutadapt software was used to perform quality control on the downstream raw data, removing the reads with joints, containing *N* (*N* indicates that the base information cannot be determined) in a proportion greater than 5%, and low quality (the number of bases with a quality value of *Q* ≤ 10 accounted for more than 20% of the whole read), to obtain the valid data (clean data). The pre-processed valid data (valid data) were then compared to the reference genome (*Bos grunniens*, genome version: v101) using the comparison software HISAT2 (13, 14). Mapped reads for each sample were assembled using StringTie (http://ccb.jhu.edu/software/stringtie/, version: stringtie-2.1.6). All transcriptomes were then merged using gffcompare software to reconstruct a comprehensive transcriptome. After the final transcriptome was generated, StringTie and ballgown were used to estimate the expression levels of all transcripts, and the expression levels of all transcripts were estimated by calculating the FPKM (Fragments per kilobase of exon model per million mapped reads, per thousand transcripts sequenced bases per million sequenced bases value, number of sequenced fragments) values were used to calculate mRNA abundance. Genes were screened in terms of both fold difference and significant level. Folds of difference FC ≥2 or FC ≤0.5 (i.e., |log_2_FC| ≥1) and *q* value <0.05 (|log_2_FC| ≥1 & *q* < 0.05) were used as the threshold criterion (no multiplicity of difference for multiple comparisons, and genes screened for *q* < 0.05 as statistically different between the multiple groups), and genes screened as a result were considered to be differentially expressed genes.

Principal component analysis (PCA) between the three groups of samples was performed using the R package vegan, and gene box plots and correlation clustering heat maps were drawn using the R package stats. The clustering heatmap was used to show the expression of genes in different samples. In order to better visualize the clustering expression pattern, log10 (FPKM+1) was used for non-biological replicates to show the gene expression. For biological replicates, the differential gene FPKM was used for gene expression display by Z-value (Zsample-i = [(FPKMsample-i)-Mean (FPKM of all samples)]/[Standard deviation (FPKM of all samples)]). Differentially expressed genes (DEGs) were enriched by GO (Gene Ontology) using the g:Profiler online website, and KEGG (Kyoto Encyclopedia of Genes and Genomes, Kyoto) was performed on DEGs using Kobas 3.0. Encyclopedia of Genes and Genomes functional analysis with a screening condition of *p* < 0.05.

### 2.4 Metabolite extraction and LC-MS detection

The samples were thawed in the ice water bath and mixed by vortices for 30 s. Then a 200 μL aliquot of each individual sample were transferred to a 1.5 mL Eppendorf tube (Supplementary Table S1). After the addition of 20 μL of ISTD solution and 400 μL of methal, the samples were vortexed for 30 s, followed by centrifuged for 10 min, 12,000 rpm [RCF = 13,800 (×g), *R* = 8.6 cm], and 4 °C. Then 360 μL of water was added in the aliquot of 300 μL supernatant, followed by vortexed for 30 s. After centrifugation (15 min, 12,000 rpm, and 4 °C), a 570 μL aliquot of the supernatant was further purified with SPE. The SPE cartridges were washed with 200 μL of methanol, then equilibrated with 200 μL H_2_O. After loading a sample, the SPE cartridges were washed with 200 μL 10% ACN/H_2_O (v/v) and 200 μL Hexane, the flow-through fraction was discarded. The cartridge was then rinsed with 24 μL 90% ACN/H_2_O (v/v). After this single-step SPE, 36 μL of water were added to the cluent. All the samples were shaked for 3 min, then the clear supernatant was subjected to UHPLC-MS/MS analysis.

Stock solutions were individually prepared by dissolving or diluting each standard substance to give a final concentration of 10 mmol/L. An aliquot of each of the stock solutions was transferred to a 10 mL flask to form a mixed working standard solution. A series of calibration standard solutions were then prepared by stepwise dilution of this mixed standard solution (containing isotopically-labelled internal standard mixture in identical concentrations with the samples).

The UHPLC separation was carried out using an ACQUITY UPLC-I/CLASS (Waters), equipped with a Waters ACQUITY UPLC BCH C8 column (100 × 2.1 mm, 1.7 μm, Waters). The mobile phase A was 0.5 mmol/L NH4F in water, and the mobile phase B was methal. The column temperature was set at 50 °C. The auto-sampler temperature was set at 10 °C and the injection volume was 10 μL. Waters Xevo TQ-S triple quadrupole mass spectrometer (Waters), equipped with an IonDrive Turbo V electrospray ionization (ESI) interface, was applied for assay development. Typical ion source parameters were: Capillary Voltages = 2.50 kV; Cone Voltages = 80 V (pos)/30 V (neg); Desolvation Temp = 550 °C; Desolvation Gas Flow = 1,100 L/Hr; Cone Gas Flow = 150 L/h; Nebuliser Gas Flow = 7.0 Bar. The MRM parameters for each of the targeted analytes were optimized using flow injection analysis, by injecting the standard solutions of the individual analytes, into the API source of the mass spectrometer. Several most sensitive transitions were used in the MRM scan mode to optimize the collision energy for each Q1/Q3 pair (Supplementary Table S2). Among the optimized MRM transitions per analyte, the Q1/Q3 pairs that showed the highest sensitivity and selectivity were selected as “quantifier” for quantitative monitoring. The additional transitions acted as “qualifier” for the purpose of verifying the identity of the target analytes. Water MassLynx V4.1 and Skyline were employed for MRM data acquisition and processing.

### 2.5 Weighted gene co-expression network analysis

**Figure 1:**
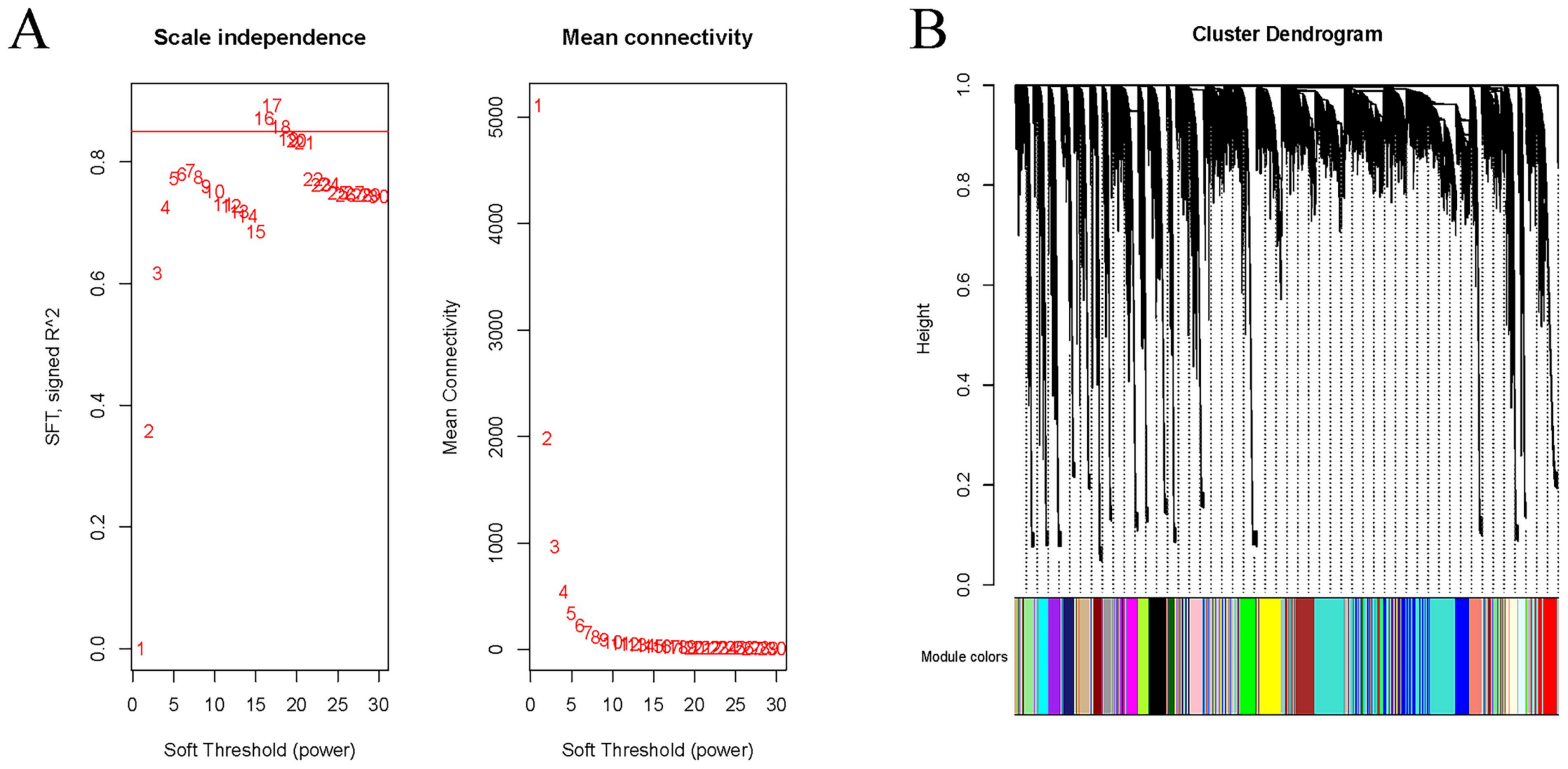
Weighted gene co-expression network analysis. **(A)** Determine the soft threshold. **(B)** Modular system clustering tree.

Weighted gene co-expression network analysis (WGCNA) target metabolomics part of the data as traits in association with the transcriptome. Based on the gene expression profiles of each breeding period, the selected gene set needs to be screened and filtered before WGCNA analysis to remove the low-quality genes that cause unstable effects on the results and to improve the accuracy of the network construction. The gene-developmental stage co-expression network was constructed using the WGCNA software package (version: 1.69). The correlation coefficients were transformed by a weighting function (adjacency function) to form an Adjacency Matrix, in which the elements are continuous. The fundamental premise is the principle of scale-free networks, which posits that the gene expression network adheres to the power function distribution characteristic of scale-free networks. The gene correlation network was made to conform to the scale-free distribution by successively weighting from 1 to 30 to find a suitable soft threshold. It was determined that the plateau threshold line set for this WGCNA analysis was 0.85 and the soft threshold was 16 (Figure 1A). To be more biologically meaningful, topological overlap measure (TOM) was used to calculate the degree of association between genes, and in addition to analysing the relationship between two genes, the connections between these two genes and other genes were also considered. The division of gene modules is based on the sparsity of connections between genes, and the TOM matrix (Similarity) is transformed into a dissimilarity matrix (Dissimilarity), which in turn constructs a hierarchical clustering tree (Figure 1B), using gene modules generated by the Dynamic Hybrid Shear Algorithm, and optimized merged gene modules [the minimum distance of merged modules (mergeCutHeight) is 0.25]. The minimum number of genes in a module (minModuleSize) was set to 30 for module detection. To identify period-specific modules, the correlation between each module and different breeding periods was explored using the WGCNA package. Modules closely associated with specific time points were screened as period-specific modules.

## 3 Results

### 3.1 Transcriptome analysis

Bipartite sequencing of 18 samples was conducted on an Illumina Novaseq^™^ 6000 platform using PE150 mode, generating 767,684,034 raw reads (average 42,649,113 per sample). Following the filtration process with Fastp, a total of 745,678,206 clean reads were obtained, constituting in excess of 97% of the original data set. Furthermore, it was observed that a minimum of 99.97% of bases exhibited quality scores of at least 20, with over 97% demonstrating scores of at least 30, and a global content of approximately 49%. The results obtained from this study suggest that the data is of a high quality and suitable for subsequent analysis. The alignment of the clean reads against the reference genome (*Bos grunniens*, v101) revealed that 85.79% were successfully mapped (Supplementary Table S3). Of these, 71% were found to be uniquely mapped to a single genomic location, while 26.05% aligned to multiple locations. The reads that were uniquely matched to a single location on the genome were used for the next data analysis.

**Figure 2:**
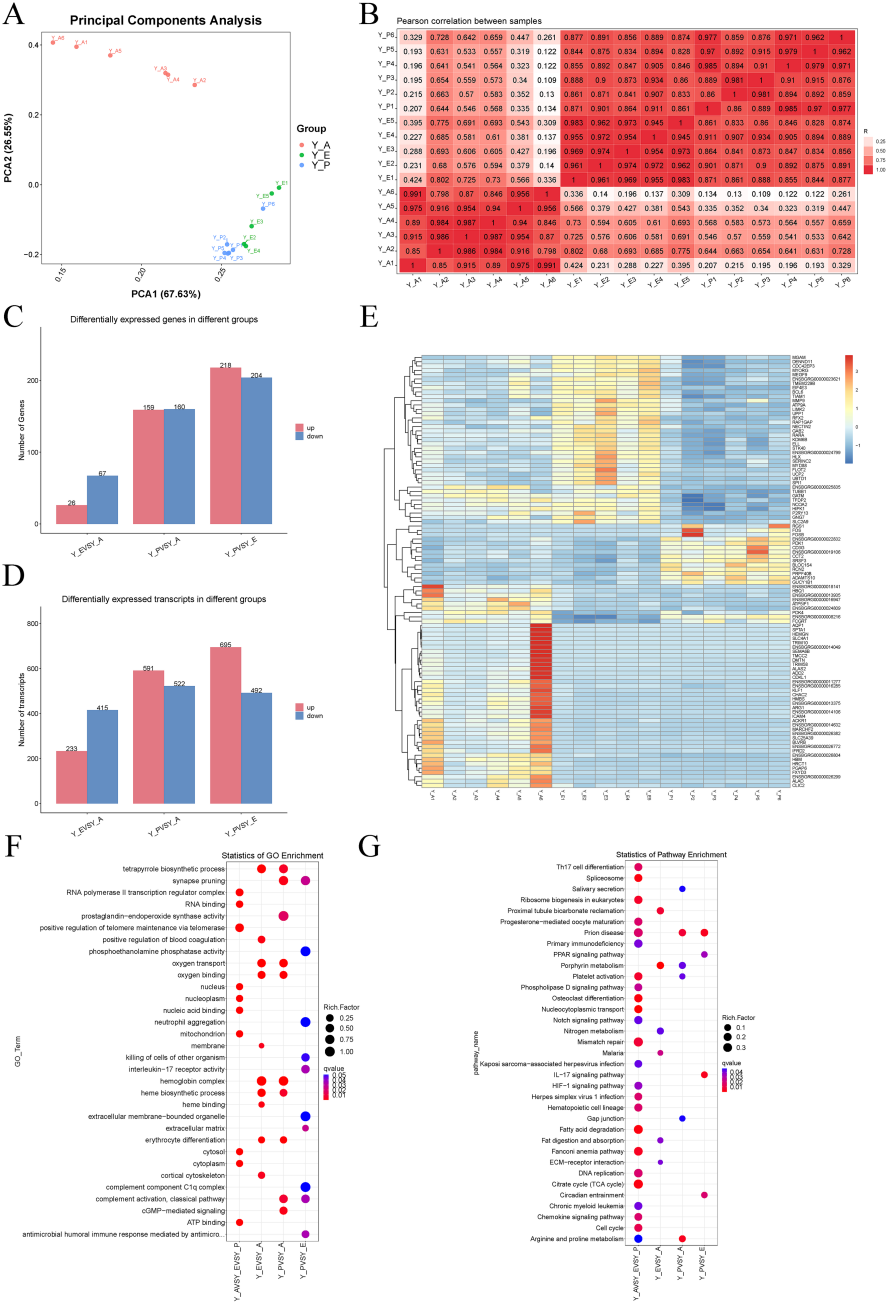
Sequencing results of yak blood transcriptome during different breeding periods. **(A)** Principal component analysis. **(B)** Correlation analysis between samples. **(C)** Differentially expressed genes. **(D)** Differentially expressed transcripts. **(E)** Cluster heat map of the top 100 differentially expressed genes. **(F)** The GO analysis of differentially expressed genes. **(G)** The KEGG analysis of differentially expressed genes.

In order to assess inter-group variability and reproducibility among the six replicates per group, principal component analysis (PCA) and correlation analysis were performed on all 18 samples. The findings of both analyses demonstrated clear separation between the three groups and high reproducibility among replicates, indicating stable and consistent results (Figures 2A,B). Gene-level expression analysis employing FPKM values (Supplementary Table S4) revealed broadly similar expression profiles across groups. The distribution of gene expression levels was quantified in the following manner: 17.27% of genes demonstrated low expression (FPKM <0.3), 51.99% exhibited moderate expression (0.3 ≤ FPKM < 15), 22.33% displayed intermediate expression (15 ≤ FPKM < 60), and 8.41% were highly expressed (FPKM ≥60). In summary, approximately half of the detected genes demonstrated moderately high expression levels.

The Y-A, Y-E and Y-P groups were divided into three comparison groups: Y-E vs. Y-A, Y-P vs. Y-E and Y-P vs. Y-A. Differentially expressed genes and transcripts were screened using |log_2_FC| ≥1 & *q* < 0.05 as threshold criteria (Figures 2C,D). The volcano diagram illustrates the screened DEGs and their overall distribution (Supplementary Figure S1). Specific analyses showed that a total of 93 DEGs were identified between the Y-E and Y-A group, with 26 up-regulated and 67 down-regulated. Three hundred and nineteen DEGs were identified in the analyses of the Y-P and Y-A group, and 159 DEGs were identified. DEGs, 159 with up-regulated expression and 160 with down-regulated expression. In contrast, a total of 422 DEGs were identified in the comparison of Y-P and Y-E group, with 218 up-regulated and 204 down-regulated. In order to characterise the expression patterns that were observed across the physiological stages, the top 100 most significant differentially expressed genes (lowest *q*-value) from each comparison were subjected to hierarchical clustering (Figure 2E). The resulting heatmap revealed clear sample clustering by physiological stage and distinct gene expression profiles among groups.

GO enrichment analysis described the distribution of genes on Biological Process (BP), Cellular Component (CC) and Molecular Function (MF). The DEGs of the three control groups were analysed by GO through the g:Profiler online website (*p* < 0.05), and the detailed results are shown in Figure 2F. In terms of biological processes, DEGs in Y-E vs. Y-A group were significantly enriched in heme biosynthesis process, oxygen transport and erythrocyte differentiation; DEGs in Y-P vs. Y-A group were significantly enriched in oxygen transport, erythrocyte differentiation and cGMP-mediated signalling; DEGs in Y-P vs. Y-E group were significantly enriched in classical pathways such as synaptic pruning, antimicrobial peptide-mediated humoral immune response and complement activation and classical pathways. In terms of molecular function, DEGs from all three groups were significantly associated with oxygen binding, heme binding, iron binding and ammonium transport across membranes.

To further understand the biological functions of different genes, KEGG pathway enrichment analysis was performed to explore the biological processes involved in DEGs at three different physiological periods. The results of the analysis are presented in Figure 2G, and the screening criterion was set at *p* < 0.05. When comparing the Y-E vs. Y-A group, 93 DEGs were found to exhibit significant enrichment in 17 pathways, which were mainly focused on extracellular matrix (ECM)-receptor interactions. In the Y-P vs. Y-A group, 319 DEGs were significantly enriched in 26 pathways, such as IL-17 signalling pathway, p53 signalling pathway, oxytocin signalling pathway and NF-kB signalling pathway were significantly associated. In the Y-P vs. Y-E group, 422 DEGs were significantly enriched in 18 pathways, which were mainly related to follicular development and ovarian activities, such as PPAR signalling pathway, ECM-receptor interaction, IL-17 signalling pathway, relaxin signalling pathway and arginine biosynthesis were significantly associated. The three groups of DEGs were co-enriched into 71 signalling pathways, which were mainly related to oocyte maturation and ovulation, etc., such as progesterone-mediated oocyte maturation, HIF-1 signalling pathway, VEGF signalling pathway and PPAR signalling pathway.

### 3.2 Metabolomic analysis

The raw data contained 18 experimental samples from which 19 metabolites were extracted, and for better analysis of the data, individual metabolites were filtered and only metabolite data with no more than 50% nulls in a single group or no more than 50% nulls in all groups were retained. Missing values in the raw data were simulated, and the numerical simulation method was filled by multiplying the minimum value by a random number between (0.1, 0.5). After preprocessing 15 metabolites were retained.

**Figure 3:**
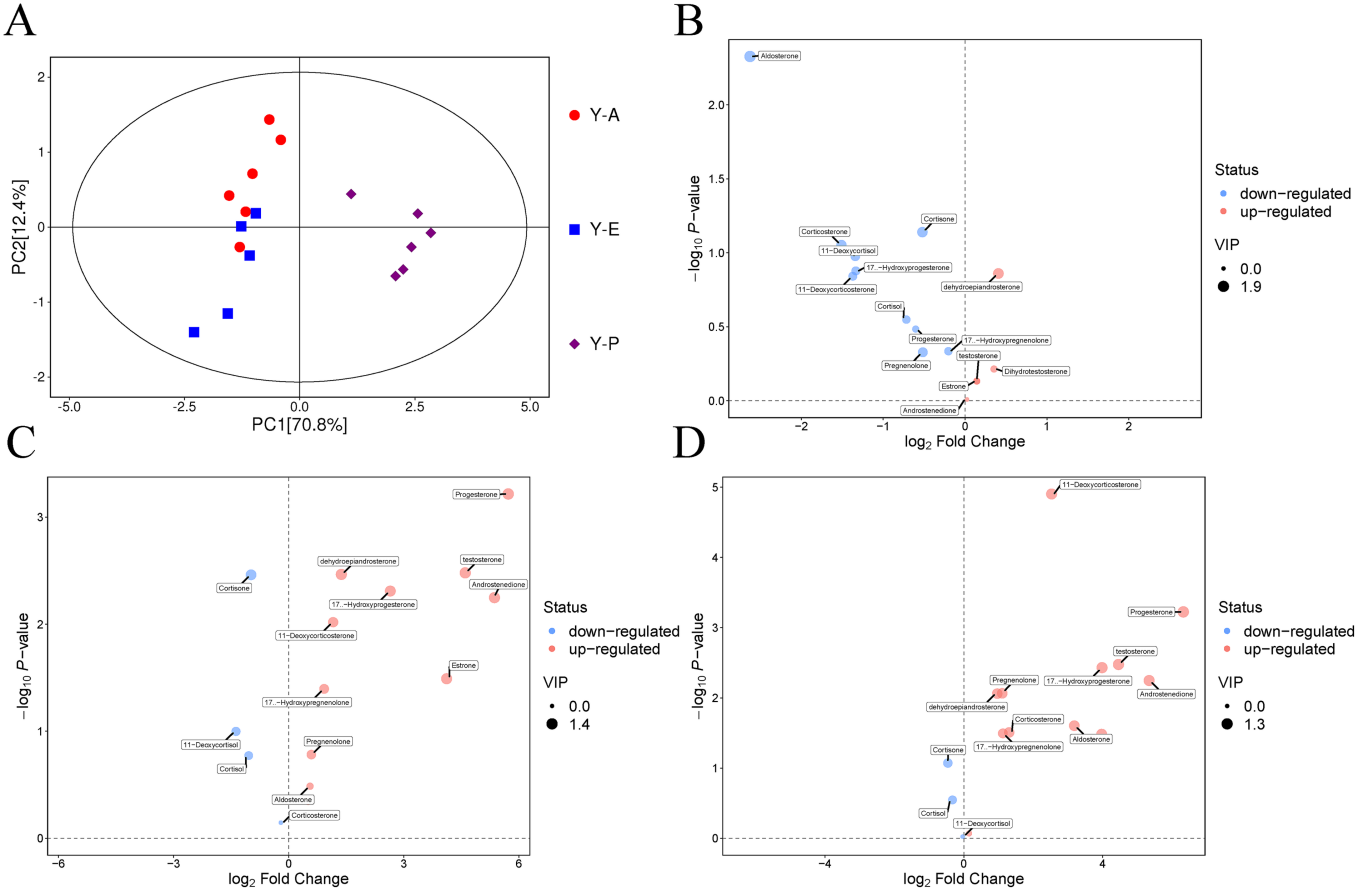
Sequencing results of yak blood metabolome at different breeding stages. **(A)** PCA score scatter plot of all samples. **(B)** Volcano plot of differentially expressed metabolites in the Y-E vs. Y-A group. **(C)** Volcano plot of differentially expressed metabolites in the Y-P vs. Y-A group. **(D)** Volcano plot of differentially expressed metabolites in the Y-P vs. Y-E group.

The PCA scores of all the samples showed that the samples within the groups could be clustered together and there was a clear trend of separation among the three groups of samples, and the variances of PC1 and PC2 were 70.8 and 12.4%, respectively (Figure 3A), which indicated that there was a difference in the metabolites in the blood of the yaks in different breeding periods. And all samples were in the 95% confidence interval, indicating that there were no outliers in the analysed samples and there was a significant trend of separation among the three groups of samples. According to the OPLS-DA (orthogonal partial least squares discriminant analysis) model, the Y-P vs. Y-A and Y-P vs. Y-E models were better than the original model, with good robustness and no overfitting phenomenon; the model of the Y-E vs. Y-A group reached an R^2^Y of 0.763 in explaining the overall variability, which indicated that the model had successfully captured the variation patterns of steroid hormone metabolites (metabolites) in different physiological stages to a certain extent. Steroid hormone metabolites at different physiological stages (Supplementary Figure S2).

For the 15 steroid hormone target metabolites after sample pretreatment, the results of statistical analyses of metabolites were visualised in the form of volcano plots (Figures 3B–D). In order to improve the reliability and biological significance of the identified differentially expressed metabolites, the metabolites retained after fulfilling the dual criteria of VIP >1 & *p* < 0.05 were considered to be the more plausible and meaningful differentially expressed metabolites after multiple screening using a combination of VIP (variable importance in projection) values and statistical significance tests. Dehydroepiandrosterone was significantly up-regulated and aldosterone, cortisone, corticosterone, 11-deoxycortisol and 17α-hydroxyprogesterone were significantly down-regulated in the Y-E vs. Y-A group; progesterone, testosterone, dehydroepiandrosterone, 17α-hydroxyprogesterone, androstenedione, 11-deoxycortisol and oestrone were significantly up-regulated and cortisone was significantly down-regulated in the Y-P vs. Y-A group; and in the Y-P vs. Y-E group 11 -deoxycorticosterone, progesterone, testosterone, 17α-hydroxyprogesterone, androstenedione, pregnenolone, dehydroepiandrosterone and aldosterone were significantly up-regulated.

### 3.3 Weighted gene co-expression network analysis

**Figure 4:**
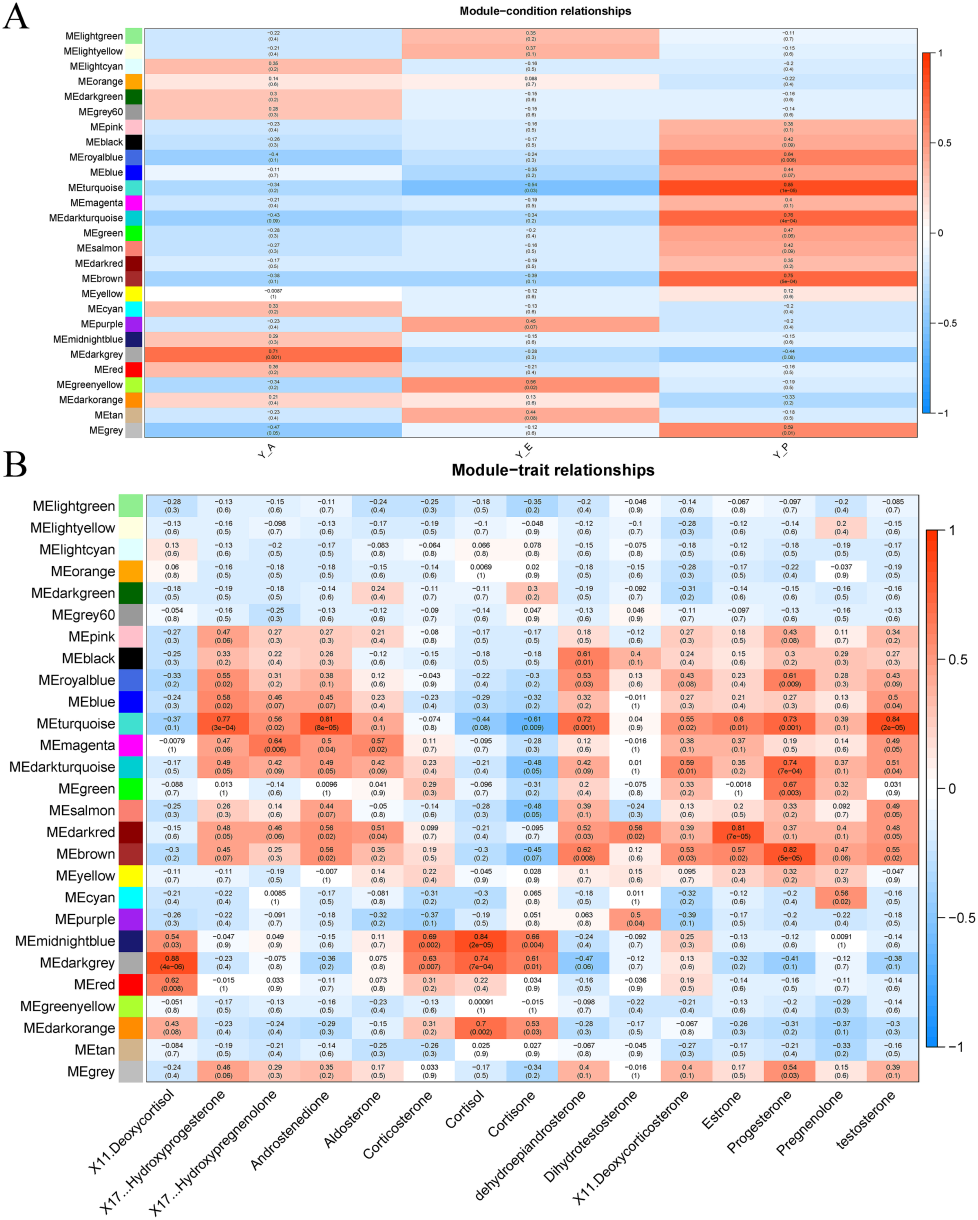
Construction of weighted gene co-expression networks. **(A)** Correlation of module conditions. **(B)** Correlation analysis of module traits.

The transcriptome sequencing results of three groups of 17 blood samples were analysed using the WGCNA approach, and 20,537 genes were finally retained for the construction of weighted gene co-expression networks. In order to find the core genes in a more biological sense, the data of steroid hormone target metabolome were jointly analysed with the transcriptome data as traits, and different breeding periods were used as developmental periods to correlate traits with gene modules, and the results of correlation strengths and weaknesses between different modules and different breeding periods were obtained, by analysing the correlation between the traits and the modules and plotting the heatmap to visually show the correlation between the modules and the given trait. We analysed the correlation between traits and modules and drew heat maps to visualize the correlation between modules and given traits. Among them, the dark grey module had the highest correlation with group Y-A (cor = 0.71, *p* = 0.001); the turquoise module had a high negative correlation with group Y-E (cor = −0.54, *p* = 0.03); and group Y-P had the highest correlation with the turquoise module (0.85, 1 × 10^−5^), dark turquoise module (cor = 0.76, *p* = 4 × 10^−4^) and brown module (cor = 0.75, *p* = 5 × 10^−4^) with a strong positive correlation (Figure 4A). Therefore, each of these four modules was used as a period-specific module with its closely associated time point for the next analysis of the biological functions of the key modules and the screening of the core (Hub) genes. Dark grey module had a high correlation with 11-deoxycortisol, corticosterone, cortisol, and cortisone in the glucocorticoid family; turquoise module and brown module had high correlation with 17α-hydroxyprogesterone, androstenedione, dehydroepiandrosterone, luteinising hormone and testosterone in the steroid hormones (Figure 4B).

**Figure 5:**
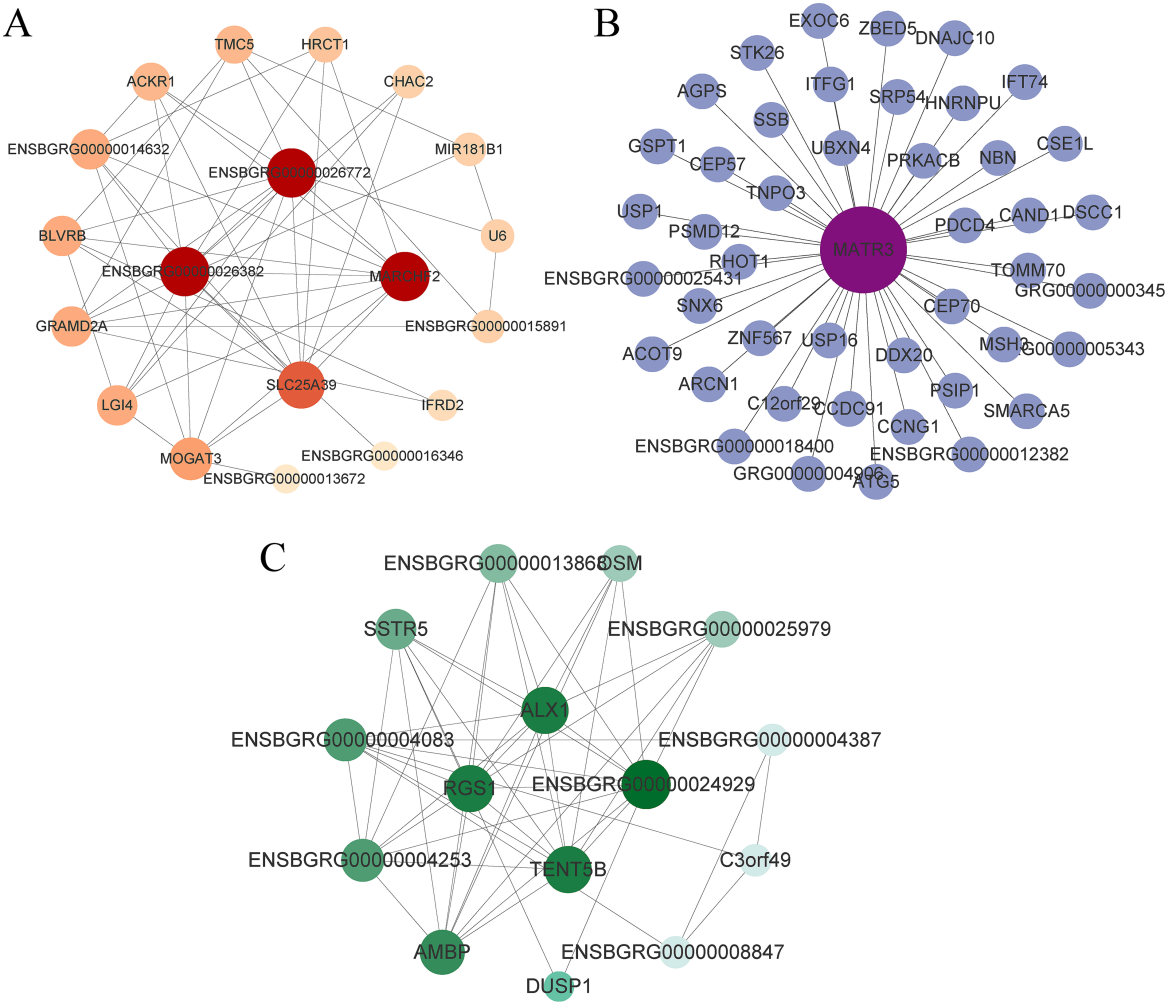
Network relationship diagram of genes in different modules. **(A)** Network diagram of genes in dark grey module. **(B)** Network diagram of genes in turquoise module. **(C)** The network diagram of the gene after the combination of turquoise, dark turquoise and brown modules.

In order to further explore the potential regulatory relationships among genes in the period-specific modules and to mine the key regulatory genes in different periods, gene co-expression networks of highly correlated modules in different breeding periods were generated using the WGCNA approach. The period-specific co-expression networks were analysed by Cytoscape software and the genes located in the top 50 connectivity nodes were visualised. In these co-expression networks, the larger the shape where the node is located indicates a higher degree of connectivity, which can be used as a Hub gene in this network. From the visualised networks, it can be seen that the *SLC25A39*, *MARCHF2*, *ENSBGRG00000026772* and *ENSBGRG00000026382* genes are Hub genes in the dark grey module (Figure 5A); the *MATR3* gene is a Hub gene in the turquoise module (Figure 5B); *ALX1*, *RGS1*, *TENT5B*, and *ENSBGRG00000024929* genes are Hub genes in the turquoise, dark turquoise, and brown modules (Figure 5C).

## 4 Discussion

The results of this study revealed that gene expression in the blood of yaks during different breeding periods is regulated by various mechanisms during ovarian and follicular development, and the regulation of genes in the signalling pathway is crucial for follicular development. Some differentially expressed genes related to oocyte meiosis and follicular development were identified in the RNA-seq results. For example, the *PDK4* gene was found to regulate oocyte meiosis in three periods of time (15), *ALAS2* and *GLP1R* genes are involved in mediating the metabolism of reproductive hormones and maintaining the balance of reproductive hormone homeostasis (16, 17), *SLC25A39* may be involved in energy metabolism in germ cells (18), *PGAP6* plays a role in germ cell growth and differentiation (19). The genes *FOS* (20), which is related to the expression of steroid hormones, and *CD36* (21), which is related to the synthesis of reproductive hormones, have been found in controls during estrus and diestrus. The *MMP9* gene is essential in ovarian development and preparation of the endometrium for fertilised egg implantation, and the *MMP9* gene was up-regulated during estrus and significantly down-regulated in gestation and estrus controls (*p* < 0.05), suggesting involvement in estrus as well as trophoblast cell differentiation in yaks (22). Involvement of the *BCL-6* gene in the oocyte meiotic pathway (23), and regulation of yak oocytes by the RARA gene to promote yak oocyte maturation were found in gestational and estrous controls (24).

The GO analysis showed that “binding” is the most important molecular function, and binding is usually the basis of various biochemical reactions and signalling in organisms, which is essential for maintaining normal physiological functions. The three groups of DEGs were significantly correlated with oxygen binding, heme binding, iron ion binding and ammonium transmembrane transport. In ovarian function, oxygen supply is essential for follicular development, especially oocyte maturation and ovulation (25). A number of differentially expressed genes are significantly enriched in biological processes such as oxygen transport, erythroid differentiation and cGMP-mediated signalling, which are indirectly linked to the physiological activities of the ovarian oocyte by influencing oxygen supply, hormone regulation and intracellular signalling. cGMP-mediated signalling pathways have been shown to influence vasodilatation and blood flow in the ovary, which in turn regulate follicle nutrient supply, waste elimination and oocyte maturation (26).

KEGG analysis showed that DEGs in all three control groups were significantly associated with progesterone-mediated oocyte maturation, the HIF-1 signalling pathway, the VEGF signalling pathway, and the PPAR signalling pathway, etc. HIF-1 (hypoxia inducible factor 1) can regulate the expression of pro-angiogenic factors (such as VEGF) to promote ovarian blood flow supply and neoangiogenesis, which is essential for follicle nutrient supply and maturation (27). In addition, HIF-1 may be involved in follicular growth and development by affecting hormone synthesis and secretion, and the balance between cell proliferation and apoptosis (28). Oocyte maturation and embryo development are controlled by intra-ovarian factors such as steroid hormones. Progesterone (P4), which is present in the follicular fluid, is important for normal ovarian function and oocyte maturation in mammals, and has a number of important functions during embryo development and implantation, including endometrial tolerance, embryo survival during pregnancy, and conversion of endometrial stromal cells to metaphase cells (29). DEGs in estrous and anestrous controls were significantly correlated with ECM receptor interaction pathways, suggesting that these pathways are involved in the regulation of ovarian development, ovulation, pregnancy and other physiological activities in yaks. Many reproductive physiological processes, such as follicle formation, require synthesis, remodelling and degradation of the extracellular matrix (ECM) (30), ECM receptors are associated with reproductive processes such as folliculogenesis, ovulation and implantation (31). DEGs in the control group during pregnancy and estrus, pregnancy and anestrus were significantly enriched in the relaxin signalling pathway and oxytocin signalling pathway. The expression of relaxin increased before embryo implantation, and the expression of relaxin receptor in endometrium (32). It has been found that relaxin promotes angiogenesis in early pregnancy (33). Oxytocin is synthesized and stored in the posterior pituitary by the hypothalamus, and then released into the blood. It can bind to the oxytocin receptor (oxytocin receptor, OXTR) on the target organ (such as myometrium, breast, etc.) and activate the G protein-coupled receptor signalling pathway. This activation usually triggers the opening of calcium channels and increases intracellular calcium concentration, which in turn leads to muscle contraction (such as uterine contraction during childbirth), milk discharge (lactational milk secretion) and other reproductive physiological responses during pregnancy (34).

In this study, we conducted a systematic and in-depth analysis of the blood samples of the three key stages of the yak breeding anestrus, estrus and pregnancy, focusing on the dynamic changes of steroid hormone metabolites and related gene expression, so as to explore the complex molecular regulatory mechanisms and their interrelationships in these three periods. First of all, the significant association of *SLC25A39* gene in the dark grey module has aroused deep concern and research interest in the functional attributes of *SLC25A39* gene in this special physiological stage during the critical period of anestrus, when the reproductive activity is relatively static and the body is in a critical period of recuperation and energy storage (35). During this period, the expression pattern of *SLC25A39* gene and its close association with the dark grey module suggest that this gene may play an indispensable role in regulating steroid hormone homeostasis and physiological preparation during anestrus, thus guiding us to further explore its special biological function and potential regulatory mechanism during anestrus. The mitochondrial ADP/ATP carrier encoded by *SLC25A39* plays a bridge role in cell energy metabolism (36, 37). In particular, it is closely related to the tricarboxylic acid cycle and oxidative phosphorylation process. The regulation of its expression level may directly affect the energy supply required for glucocorticoid synthesis (18, 38).

In the anestrus, the expression change of SLC25A39 may be to adjust the biosynthesis rate of glucocorticoids to achieve the appropriate hormone level required for the beginning of the next stage of estrus. Glucocorticoids play an important role in regulating immune response (39), stress response (40) and metabolism (41). The expression of *SLC25A39* in anestrus may be indirectly involved in regulating immune homeostasis, reducing non-essential stress and maintaining physiological preparation during the intermittent period of reproductive cycle. In-depth study of the spatial and temporal expression characteristics of *SLC25A39* in different tissues or cell types during anestrus and its interaction with key enzymes in glucocorticoid biosynthesis will help to reveal its precise regulatory mechanism at the beginning of the reproductive cycle. During estrus, the expression of *MARCHF2* gene and the level of dehydroepiandrosterone (DHEA) were significantly increased, revealing that they play a key role in the fluctuation of reproductive hormones and the activation of reproductive system. Estrus is a critical time when reproductive hormones fluctuate violently and the reproductive system is rapidly activated (42). *MARCHF2*, as an E3 ubiquitin ligase, may be directly involved in the ubiquitination process of hormone receptors, thereby affecting the endocytosis, degradation and functional activity of steroid hormone receptors (43). During estrus, the up-regulation of *MARCHF2* may help to regulate the turnover rate of steroid hormone receptors and ensure the effective transmission of reproductive hormone signals to adapt to the rapidly changing hormone requirements during follicular development, ovulation and corpus luteum formation. At the same time, DHEA is an important steroid hormone precursor, and its increase during estrus reflects its core position in the rapid synthesis of active sex hormones such as testosterone and estradiol. Specifically, DHEA may effectively supplement the sex hormone pool by participating in biochemical pathways such as cholesterol side chain cleavage and 17α-hydroxylation to meet the needs of reproductive activities during estrus (44, 45). In-depth study of the specific regulatory mechanism of *MARCHF2* on hormone receptors and the role of DHEA in the steroid hormone synthesis pathway will help to reveal the complexity and accuracy of the reproductive endocrine regulatory network during estrus. The down-regulation of glucocorticoid hormones such as aldosterone and cortisone during pregnancy reflects subtle adjustments in maternal immune and nutritional allocation strategies (46). These glucocorticoids are involved in the regulation of physiological processes such as salt and water balance, glycogen metabolism and inflammatory response (47). During pregnancy, appropriate reduction of glucocorticoid levels helps to prevent excessive immunosuppression and stress response, thereby maintaining the balance of maternal and fetal immune tolerance and ensuring the normal development of the fetus (48). At the same time, DHEA still maintains a high level during pregnancy, which may be because it not only acts as a precursor of sex hormones, but also indirectly supports fetal development and maintains hormone homeostasis during pregnancy by participating in the regulation of lipid metabolism, glucose metabolism, and antioxidant defense. Specifically, DHEA may have a positive impact on maternal and fetal nutritional supply and physiological homeostasis by affecting insulin sensitivity, lipid metabolism and antioxidant enzyme activity. The in-depth study of the specific functional pathways of these hormones during pregnancy will contribute to a more comprehensive understanding of the complex regulatory mechanisms of the reproductive endocrine system during pregnancy.

In summary, through the comprehensive analysis of steroid hormone metabolites and gene expression data during anestrus, estrus and pregnancy, the special roles of genes such as *SLC25A39* and *MARCHF2* in different reproductive stages were revealed, as well as the dynamic changes and correlations of hormones such as DHEA, aldosterone and cortisone in the regulation of reproductive cycle. It is recommended that future research focus on further elucidation of the specific mechanisms by which these genes and hormones function within reproductive regulatory networks. Such research should include an analysis of their upstream regulatory elements, downstream effector targets, and synergistic or antagonistic relationships with other reproductive hormones. Subsequent research endeavours will concentrate on the functional validation of key candidate genes. This will be achieved by identifying and validating blood biomarkers for early pregnancy or estrus detection, based on metabolomic and gene expression profiling. This study will greatly enrich and improve the theoretical system of yak reproductive biology, and provide a more solid scientific basis for improving the reproductive performance of yak, formulating effective reproductive management strategies, and preventing and treating reproductive disorders. At the same time, these research results may also bring reference and inspiration for the study of reproductive biology of other mammals.

## 5 Conclusion

In this study, by analysing the changes of steroid hormone metabolism and gene expression in blood samples at different stages of yak breeding cycle, DEGs (*PDK4*, *ALAS2*, *GLP1R*, *SLC25A39*, *PGAP6*, *FOS*, *CD36*, *MMP9*, *BCL-6*, etc.) were identified. It plays a key role in ovarian function, follicular development, hormone homeostasis and energy metabolism. Functional annotation and enrichment analysis showed that DEGs were involved in ovarian angiogenesis, hormone synthesis and follicular development. It is revealed that *SLC25A39* is involved in the regulation of energy metabolism and glucocorticoid homeostasis during anestrus. *MARCHF2* and DHEA play a role in reproductive hormone fluctuation and system activation during estrus. Glucocorticoids and DHEA during pregnancy play a key role in maintaining hormone homeostasis and immune tolerance.

## Data Availability

The datasets presented in this study can be found in online repositories. The names of the repository/repositories and accession number(s) can be found at: https://www.ncbi.nlm.nih.gov/, GSE282450.

## Supplementary material

SUPPLEMENTARY TABLE S1 Quantification results-UHPLC-QQQ-MS.

SUPPLEMENTARY TABLE S2 MRM parameters-UHPLC-QQQ-MS.

SUPPLEMENTARY TABLE S3 Mapping results with yak reference genome.

SUPPLEMENTARY TABLE S4 Gene expression levels measured by FPKM in yak blood.
