# Supplementary material for: Analysis of transcriptome and metabolome characteristics of blood in yaks at different reproductive stages
Source: Front Vet Sci. 2025 Oct 17;12:1633877. doi: 10.3389/fvets.2025.1633877 (PMC12575335; doi:10.3389/fvets.2025.1633877)
Supplement: Supplementary file 5 [file Image_1.pdf]

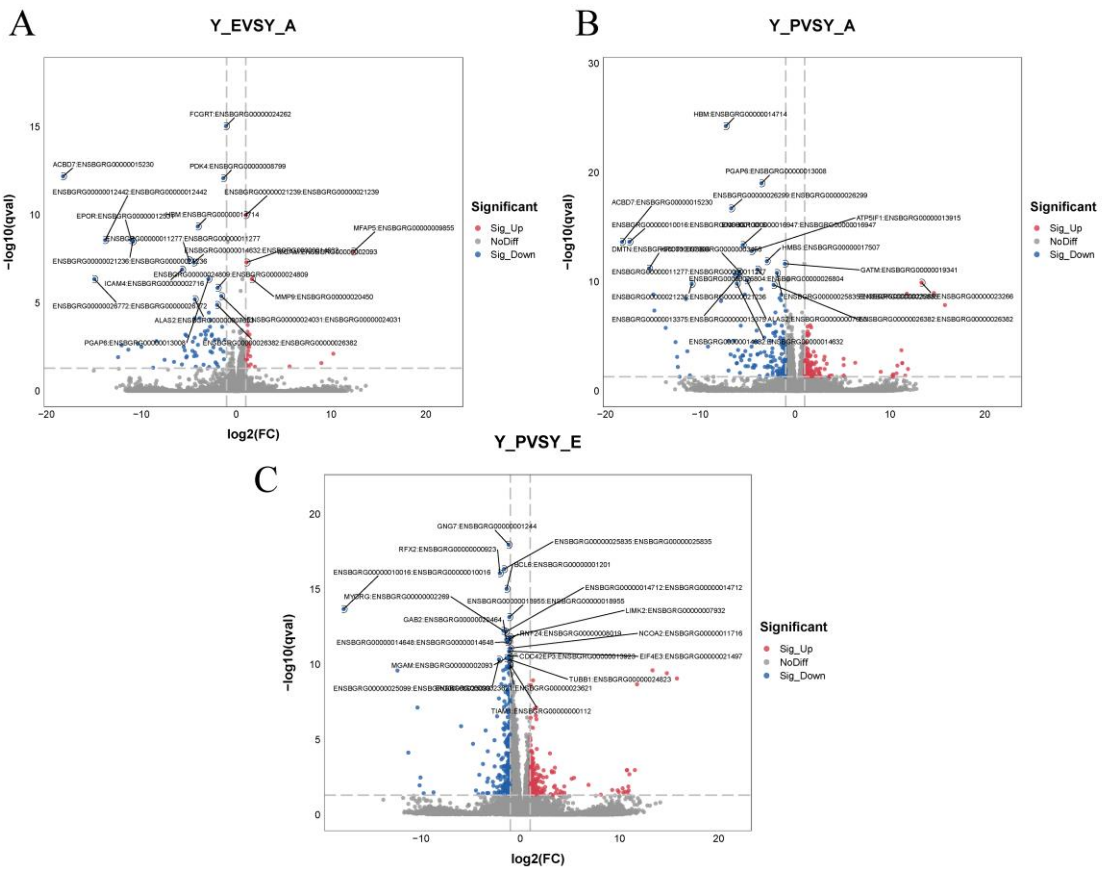

Figure. S1. Volcano map of differentially expressed genes. (A) Y-E vs Y-A, (B) Y-P vs Y-A (C) Y-P vs Y-E.

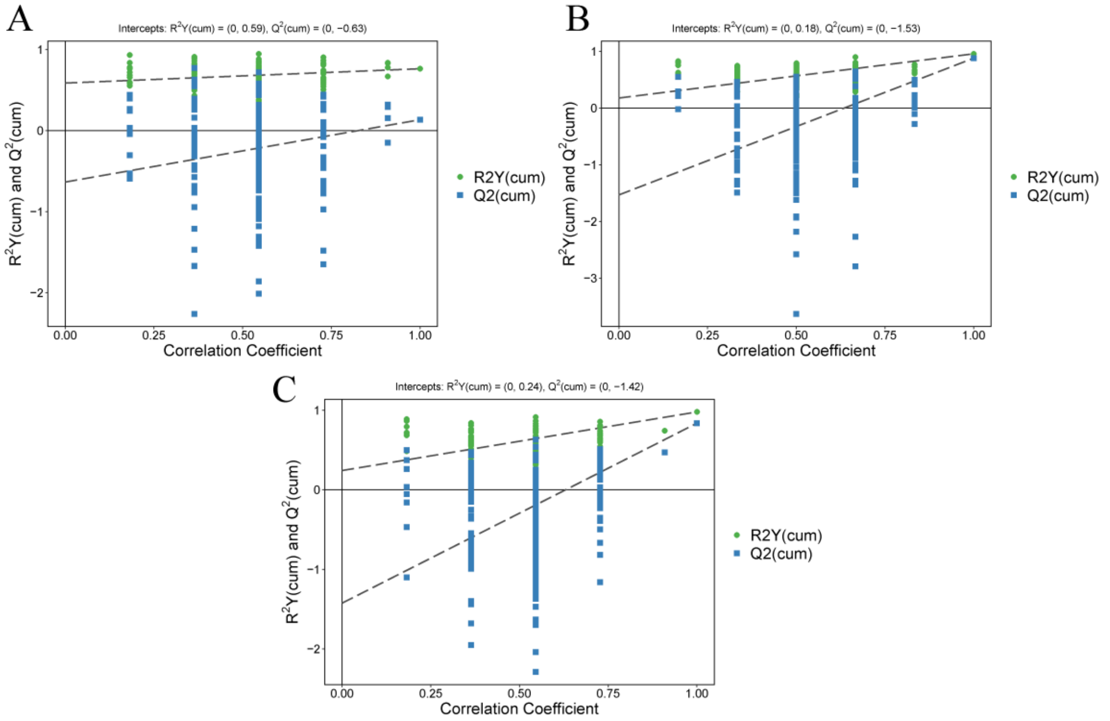

Figure. S2. Permutation test of OPLS-DA model. (A) Y-E vs Y-A, (B) Y-P vs Y-A (C) Y-P vs Y-E.
